# Author Correction: The structure of the ubiquitin-like modifier FAT10 reveals an alternative targeting mechanism for proteasomal degradation

**DOI:** 10.1038/s41467-018-07183-0

**Published:** 2018-11-02

**Authors:** Annette Aichem, Samira Anders, Nicola Catone, Philip Rößler, Sophie Stotz, Andrej Berg, Ricarda Schwab, Sophia Scheuermann, Johanna Bialas, Mira C. Schütz-Stoffregen, Gunter Schmidtke, Christine Peter, Marcus Groettrup, Silke Wiesner

**Affiliations:** 1 Division of Immunology, Department of Biology, University of Konstanz, Konstanz D-78457, Germany; 2 Biotechnology Institute Thurgau at the University of Konstanz, Kreuzlingen CH-8280, Switzerland; 3 Max Planck Institute for Developmental Biology, Tübingen D-72076, Germany; 4 Computational and Theoretical Chemistry, Department of Chemistry, University of Konstanz, Konstanz D-78457, Germany; 5 Institute of Biophysics and Physical Biochemistry, University of Regensburg, Regensburg D-93040, Germany

Correction to: *Nature Communication* 10.1038/s41467-018-05776-3; published online 20 August 2018

The original version of the Supplementary Information associated with this Article inadvertently omitted Supplementary Table 3. The HTML version of the Article has been updated to include a corrected version of the Supplementary Information.

