# Supplementary material for: The structure of the ubiquitin-like modifier FAT10 reveals an alternative targeting mechanism for proteasomal degradation
Source: Nat Commun. 2018 Aug 20;9:3321. doi: 10.1038/s41467-018-05776-3 (PMC6102260; doi:10.1038/s41467-018-05776-3)
Supplement: Supplementary file 1 — Supplementary Information [file 41467_2018_5776_MOESM1_ESM.pdf]

The structure of the ubiquitin-like modifier FAT10 reveals an alternative targeting mechanism for proteasomal degradation

A. Aichem et al.

Supplementary Information

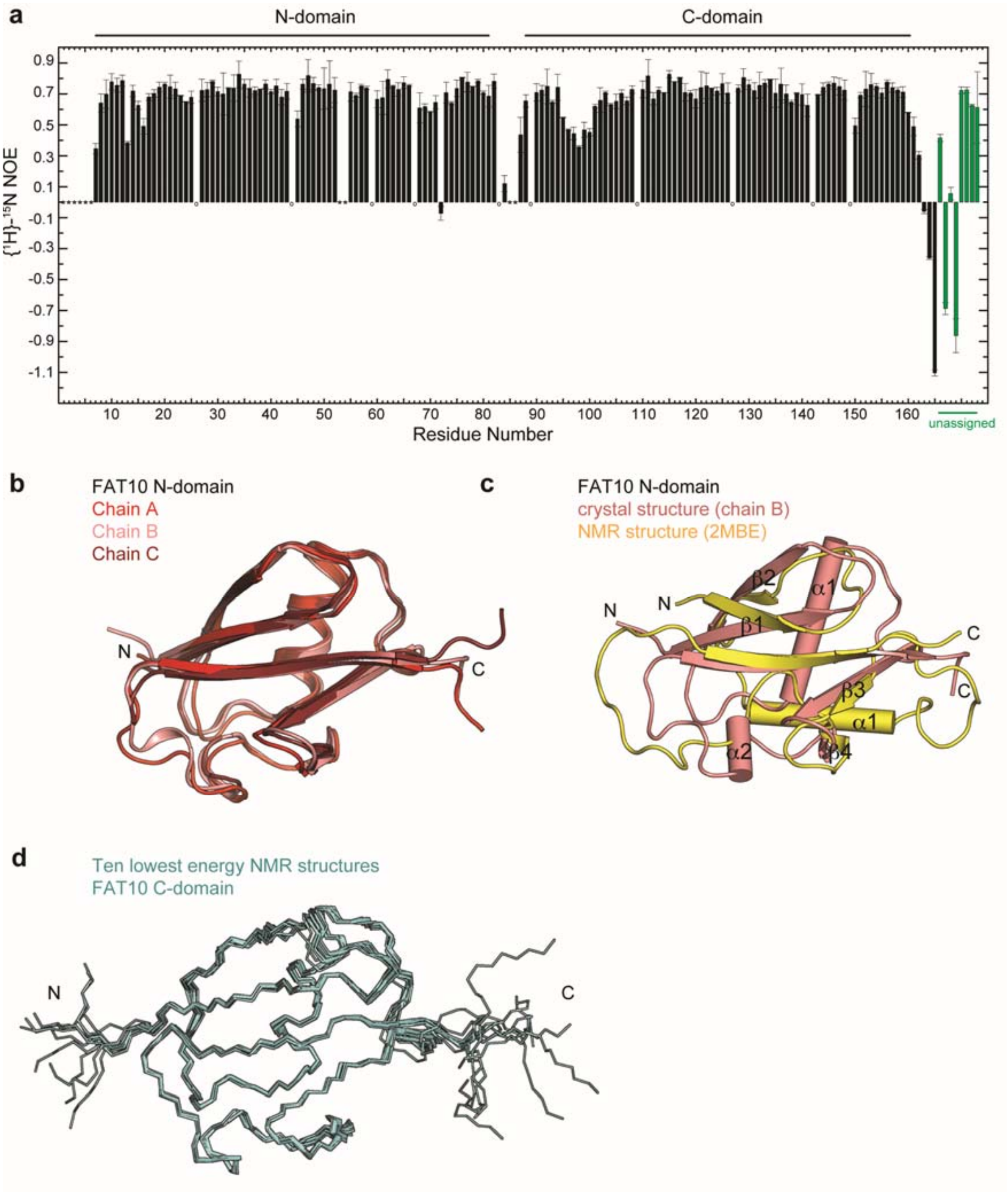

Supplementary Figure 1 Structural analysis of full-length FAT10 and the two FAT10 UBDs. (**a**) Steady-state {^{1}H}-^{15}N NOE values of full-length FAT10 plotted as means +/- standard deviations. Unassigned residues are indicated with asterisks and prolines with open circles. {^{1}H}-^{15}N NOE values for unassigned peaks are colored in green. Low {^{1}H}-^{15}N NOE values indicate elevated backbone flexibility. (**b**) Overlay of the three FAT10 N-domain protein chains (A, B, C) in the asymmetric unit. (**c**) Overlay of the crystal structure (chain B) of the FAT10 N-domain determined in this study (salmon) with the previously determined NMR

structure of this domain (yellow; PDB-ID: 2MBE)[1]. (**d**) Superposition of the backbone traces of the ten lowest energy NMR structures of the FAT10 C-domain determined in this study.

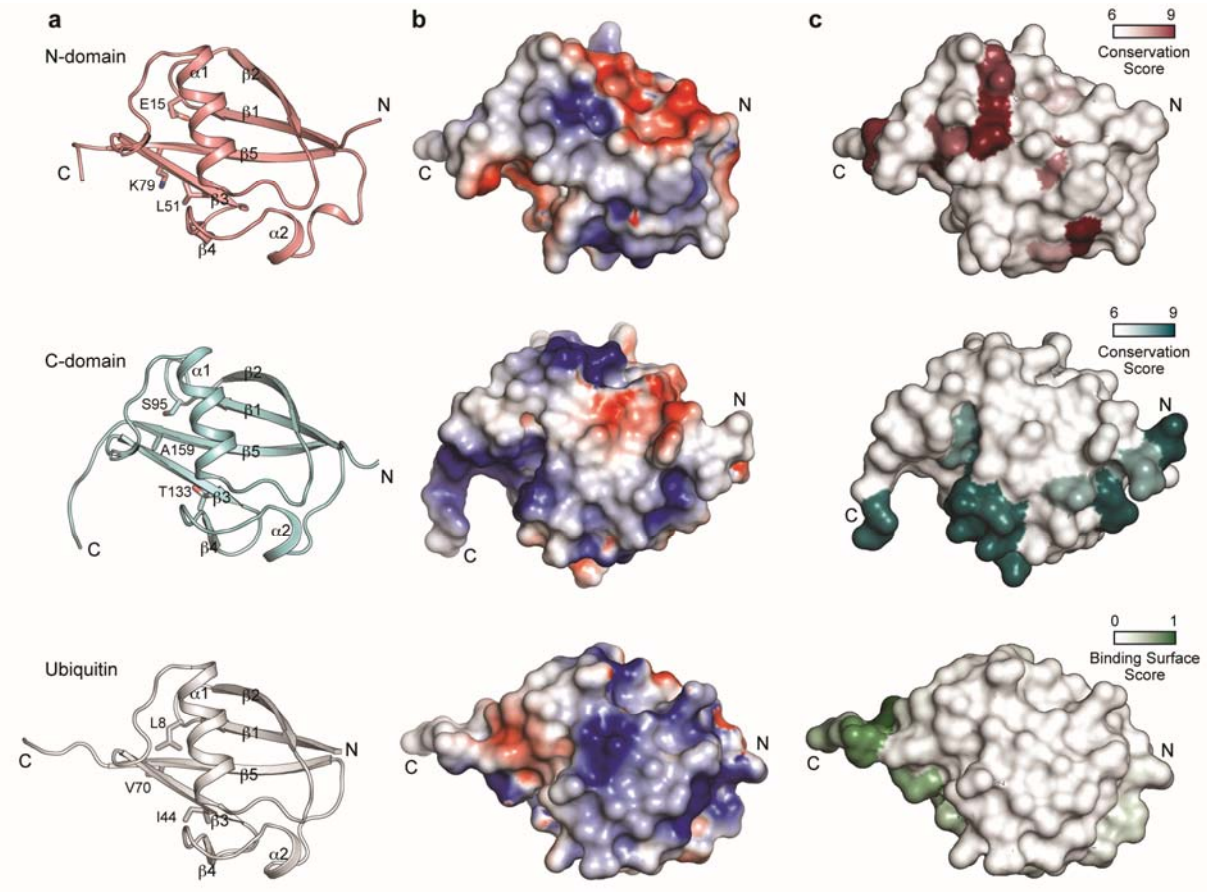

Supplementary Figure 2 The two FAT10 UBDs and Ub have distinct surface properties. (**a**) Ribbon representation of the FAT10 N- (top) and C- (center) domain and Ub (bottom). Side-chains equivalent to the hydrophobic patch (L8, I44 and V70) in Ub are shown as sticks. (**b**) Electrostatic surface potentials of the FAT10 N- (top) and C-domains (center) and Ub (bottom). Negatively charged areas are colored in red, while positively charged regions are in blue and neutral residues in white. (**c**) Surface plots of the FAT10 N- (red; top) and C-domains (teal; center) colored by conservation of residues in mammalian FAT10 proteins. The surface of Ub is color coded in green by occurrence of residues at the interfaces of Ub-protein complexes (bottom). All structure and surface representations are rotated by 180° with respect to the orientation shown in **Fig. 1d-f** and **Fig. 2b-d**.

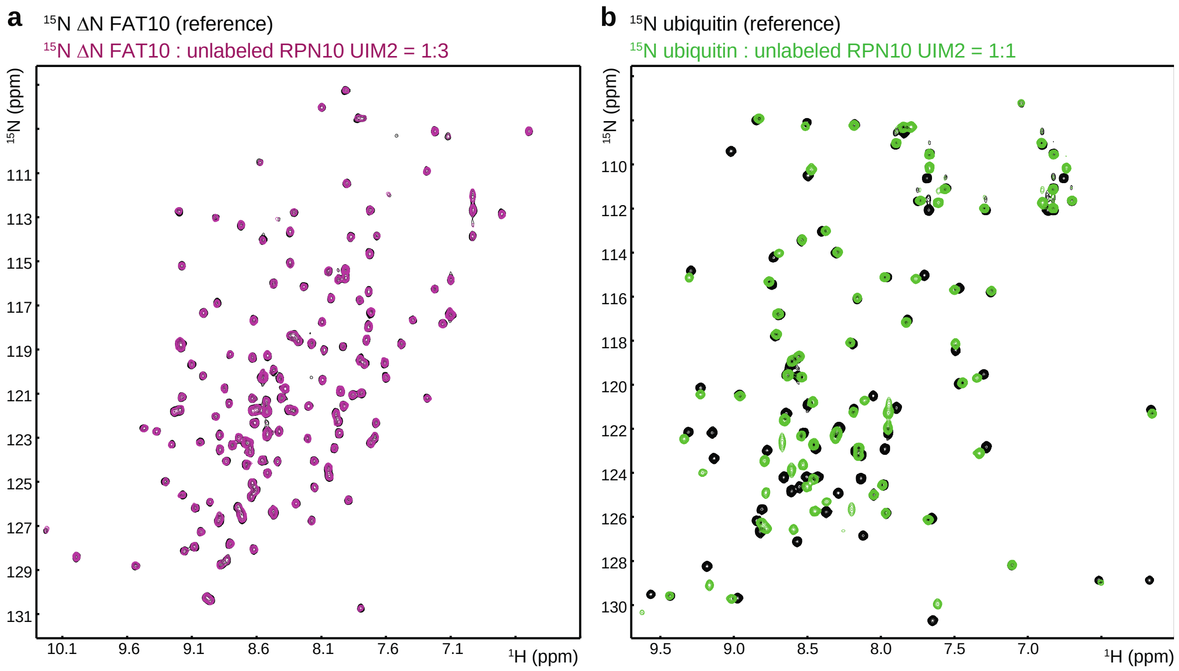

Supplementary Figure 3 The two FAT10 UBDs and Ub have distinct binding specificities. (**a**) Overlay of the ^{1}H,^{15}N-correlation spectrum of ^{15}N-labeled ΔN FAT10 in the absence (black) or presence (magenta) of a three-fold stoichiometric excess of unlabeled RPN10 UIM2. (**b**) As (a), but for ^{15}N-labeled Ub in the absence (black) or presence (green) of an equimolar amount of unlabeled RPN10 UIM2. In contrast to Ub, the FAT10 domains display no chemical shift changes upon addition of the RPN10 UIM2. This demonstrates that the FAT10 ubiquitin-like domains (UBDs) have binding specificities that are distinct from Ub.

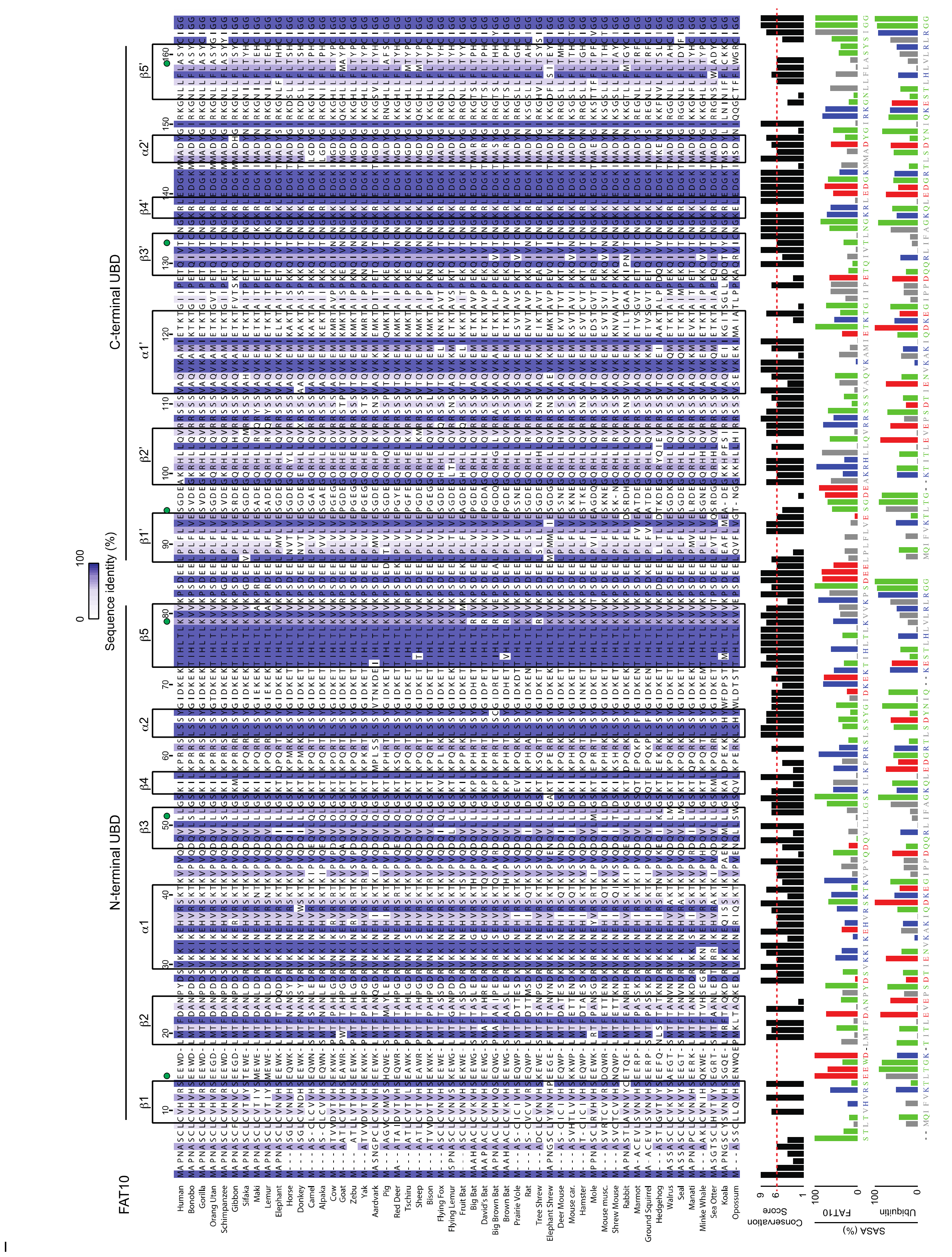

Supplementary Figure 4 Sequence conservation in mammalian FAT10 proteins. FAT10 protein sequences are color coded from white to dark-blue by sequence identity. Secondary

structure elements are boxed. Green circles indicate the positions equivalent to the hydrophobic patch in Ub (L8, I44 abd V70). The conservation score and solvent accessible surface areas (SASA) were calculated with the ConSurf server and ASA-View using the structures determined in this study and PDB-ID: 1UBQ for Ub. The red dashed line for the conservation score indicates the cut-off used for color-coding the protein surfaces in **Fig. 2d** and **Supplementary Fig. 2c**. Red, blue, green and gray bars and letters in the SASA plots indicate negatively charged, positively charged, hydrophilic, and hydrophobic residues, respectively. The following database entries were used for the sequence alignment: AAD52982.1 (human), XP_003829773.1 (bonobo), XP_004043579.1 (gorilla), XP_002816657.1 (orang utan)_ XP_527322.2 (schimpanzee), XP_003272054.1 (gibbon), XP_012515562.1 (sifaka), XP_012615813.1 (maki), XP_012615813.1 (lemur), XP_003421855.1 (elephant), XP_005603715.1 (horse), XP_014723452.1 (donkey), XP_010972404.1 (camel), XP_015104261.1 (alpaka), NP_001193402.1 (cow), XP_005696712.2 (goat), XP_019841843.1 (zebu), XP_005911568.1 (yak), XP_007949628.1 (aardvark), NP_001153560.1 (pig), OWK13121.1 (red deer), XP_005965519.1 (tschiru), XP_004019060.1 (sheep), XP_010830873.1 (bison), XP_011385823.1 (flying fox), XP_008589165.1 (flying lemur), XP_015976219.1 (fruit bat), XP_005853121.1 (big bat), XP_006759175.1 (David's bat), XP_008155616.1 (big brown bat), XP_006107997.1 (brown bat), XP_005360544.1 (prairie vole), Q921A3.1 (rat), XP_006160364.1 (tree shrew), XP_00689630 (elephant shrew), XP_006998398.1 (deer mouse), XP_021005770.1 (mouse car.; mus caroli), XP_005087259.1 (hamster), XP_004694777.2 (mole), NP_075626.1 (mouse musc.; mus musculus), XP_021074154.1 (shrew_mouse), XP_002714381.2 (rabbit), XP_015362966.1 (marmot), XP_005338891.1 (ground squirrel), XP_007532384.2 (hedgehog), XP_004411346.1 (walrus), XP_006748804.1 (seal), XP_004387158.1 (manati), XP_007179150.1 (minke whale), XP_022348403.1 (sea otter), XP_020829714.1 (koala), XP_003340419.2 (opossum)

a

```
KPSDE          1 mghhhhhhgsdykdhdgdykdhdidykddddkefmapnasclcvhvrseewdlmtfdanpydsvkkikehvrsktkvpvq
5x Ala         1 mghhhhhhgsdykdhdgdykdhdidykddddkefmapnasclcvhvrseewdlmtfdanpydsvkkikehvrsktkvpvq
5x Pro         1 mghhhhhhgsdykdhdgdykdhdidykddddkefmapnasclcvhvrseewdlmtfdanpydsvkkikehvrsktkvpvq
5x Gly         1 mghhhhhhgsdykdhdgdykdhdidykddddkefmapnasclcvhvrseewdlmtfdanpydsvkkikehvrsktkvpvq
delta linker   1 mghhhhhhgsdykdhdgdykdhdidykddddkefmapnasclcvhvrseewdlmtfdanpydsvkkikehvrsktkvpvq

KPSDE         81 dqvlllgskilkprrslssygidkektihltlkvvkpsdeelplflvesgdeakrhllqvrrsssvaqvkamietktgii
5x Ala        81 dqvlllgskilkprrslssygidkektihltlkvvaaaaaelplflvesgdeakrhllqvrrsssvaqvkamietktgii
5x Pro        81 dqvlllgskilkprrslssygidkektihltlkvvpppppelplflvesgdeakrhllqvrrsssvaqvkamietktgii
5x Gly        81 dqvlllgskilkprrslssygidkektihltlkvvgggggelplflvesgdeakrhllqvrrsssvaqvkamietktgii
delta linker  81 dqvlllgskilkprrslssygidkektihltlkvv-----elplflvesgdeakrhllqvrrsssvaqvkamietktgii

KPSDE        161 petqivtcngkrledgkmmadygirkgnllflasycigg
5x Ala        161 petqivtcngkrledgkmmadygirkgnllflasycigg
5x Pro        161 petqivtcngkrledgkmmadygirkgnllflasycigg
5x Gly        161 petqivtcngkrledgkmmadygirkgnllflasycigg
delta linker 156 petqivtcngkrledgkmmadygirkgnllflasycigg
```

b

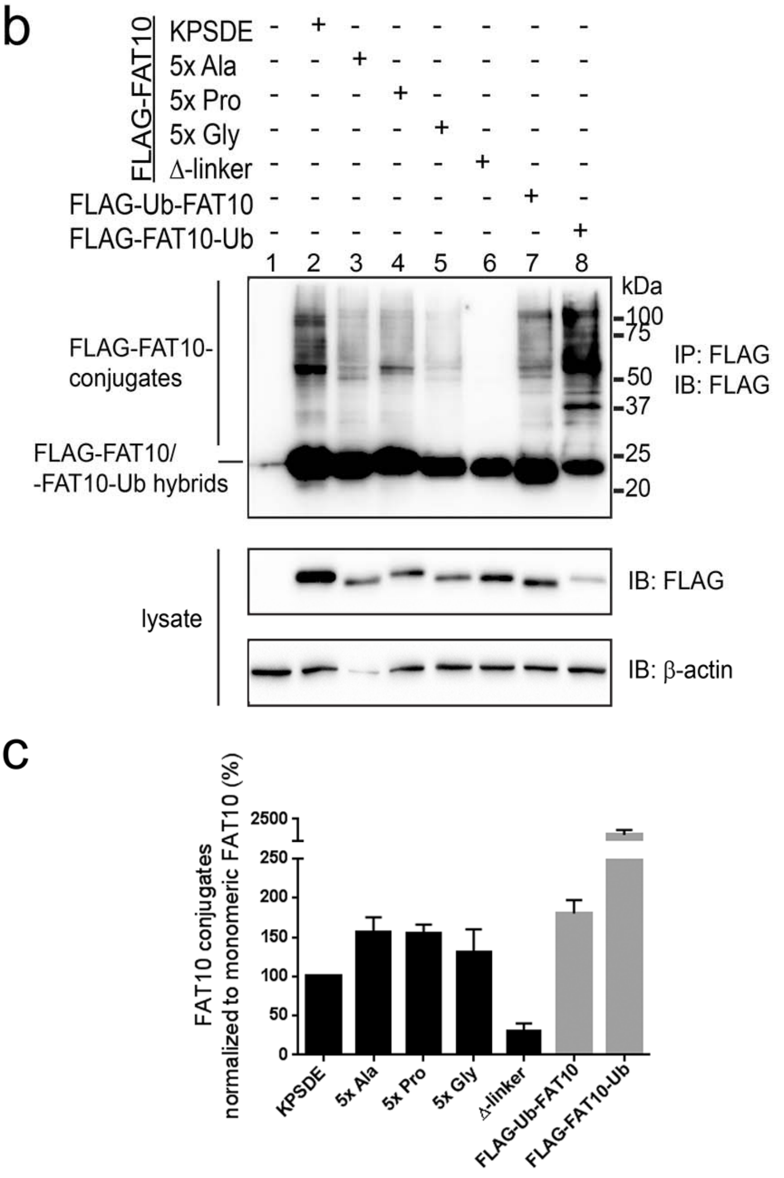

Supplementary Figure 5 The FAT10 linker is required for conjugate formation. (a) Shown is the alignment of the deduced amino acid sequences of FAT10 containing the wildtype linker sequence KPSDE as well as the different linker mutants 5x Ala, 5x Pro, 5x Gly and Δlinker. Similar amino acids are highlighted in green, the sequence of the His-3xFLAG tag is underlined. **(b)** HEK293 cells were transiently transfected with expression constructs for the different FAT10 variants, as indicated. 24 hours after transfection, cells were directly lysed in denaturing lysis buffer, renatured in RIPA buffer and subjected to immunoprecipitation using EZview Red Anti-FLAG-M2 Affinity Gel. Proteins were separated on 12.5% Laemmli gels. The conjugation of wildtype as well as of mutated FAT10 to its substrates was analyzed by western blotting using an antibody reactive against FLAG. β-actin was used as loading control. Shown is one representative experiment out of three experiments with similar outcomes. (**c**) Graphic depiction of the ECL signals of the immunoprecipitated FLAG-FAT10 conjugates from (b). Enhanced chemiluminescence (ECL) signals of FAT10 conjugates of three independent experiments were quantified and normalized to the ECL signals of the respective monomeric FAT10 in the lysate. The value of wildtype FAT10 conjugates normalized to the respective FAT10 in the lysate (KPSDE, lane 2) was set to unity and the other values were calculated accordingly. The mean of three independent experiments with similar outcomes is shown as means ± s.e.m.

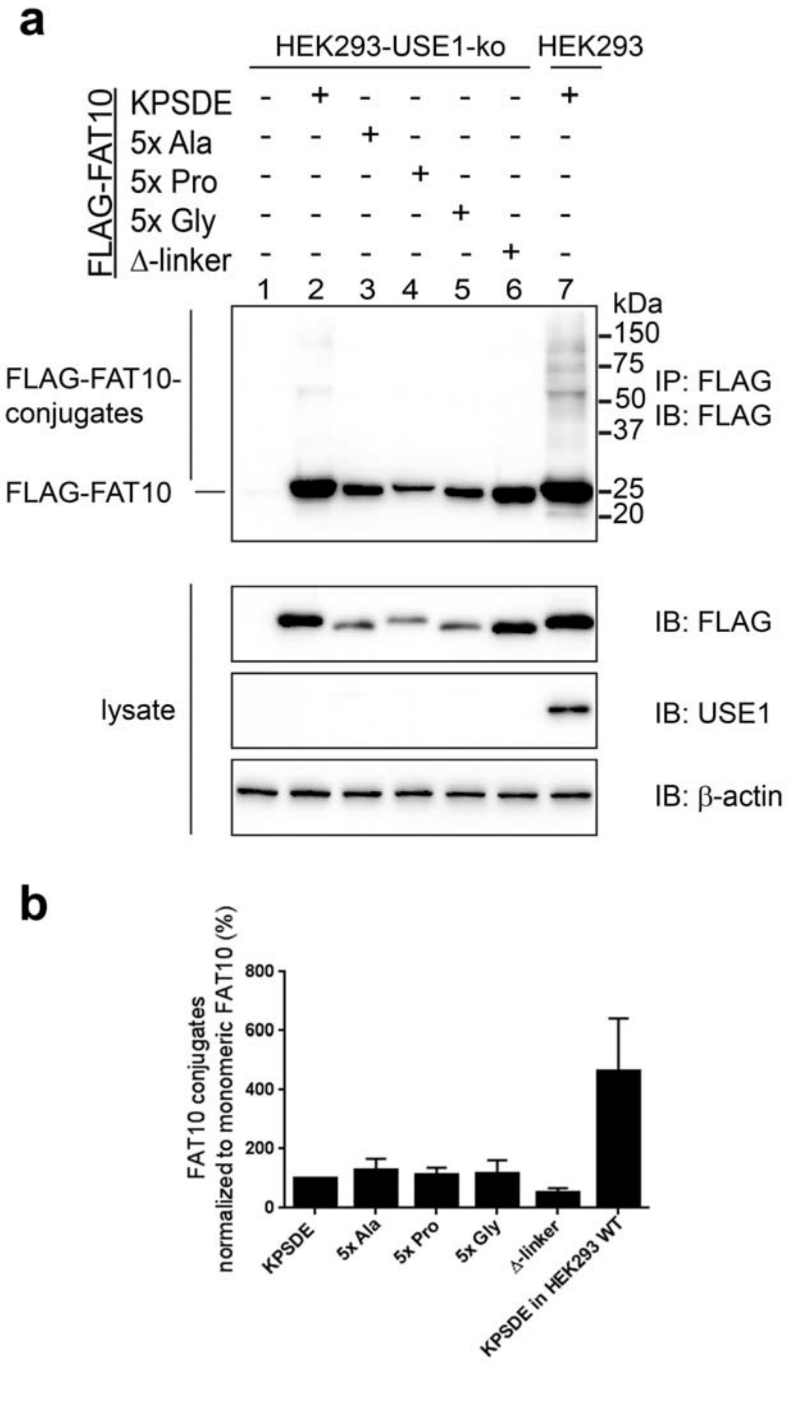

Supplementary Figure 6 FAT10 linker mutants in USE1-deficient cells confirm USE1 as E2 enzyme. (**a**) HEK293 wildtype or HEK293-CRISPR USE1-knockout cells were transiently transfected with expression constructs for the different FAT10 variants, as indicated. 24 hours after transfection, cells were harvested, lysed and subjected to immunoprecipitation and western blot analysis. Proteins were separated on 12.5% Laemmli gels and analyzed by western blotting

with a monoclonal antibody reactive against FLAG or a polyclonal antibody reactive against USE1. β-actin was used as loading control. One representative experiment out of three experiments with similar outcomes is shown. (**b**) Graphic depiction of the enhanced chemiluminscence (ECL) signals of the immunoprecipitated FLAG-FAT10 conjugates from (a). ECL signals of FAT10 conjugates of three independent experiments were quantified and normalized to the ECL signals of the respective monomeric FAT10 in the lysate. The value of wildtype FAT10 conjugates expressed in HEK293-CRISPR USE1-knockout cells normalized to the respective FAT10 in the lysate (KPSDE, lane 2) was set to unity and the other values were calculated accordingly. The mean of three independent experiments with similar outcomes is shown as means ± s.e.m.

a

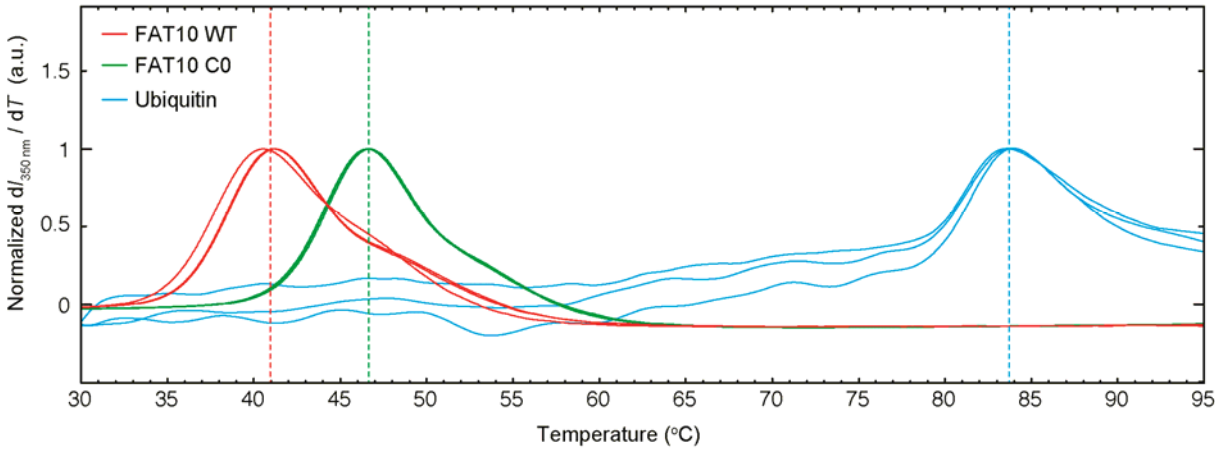

b

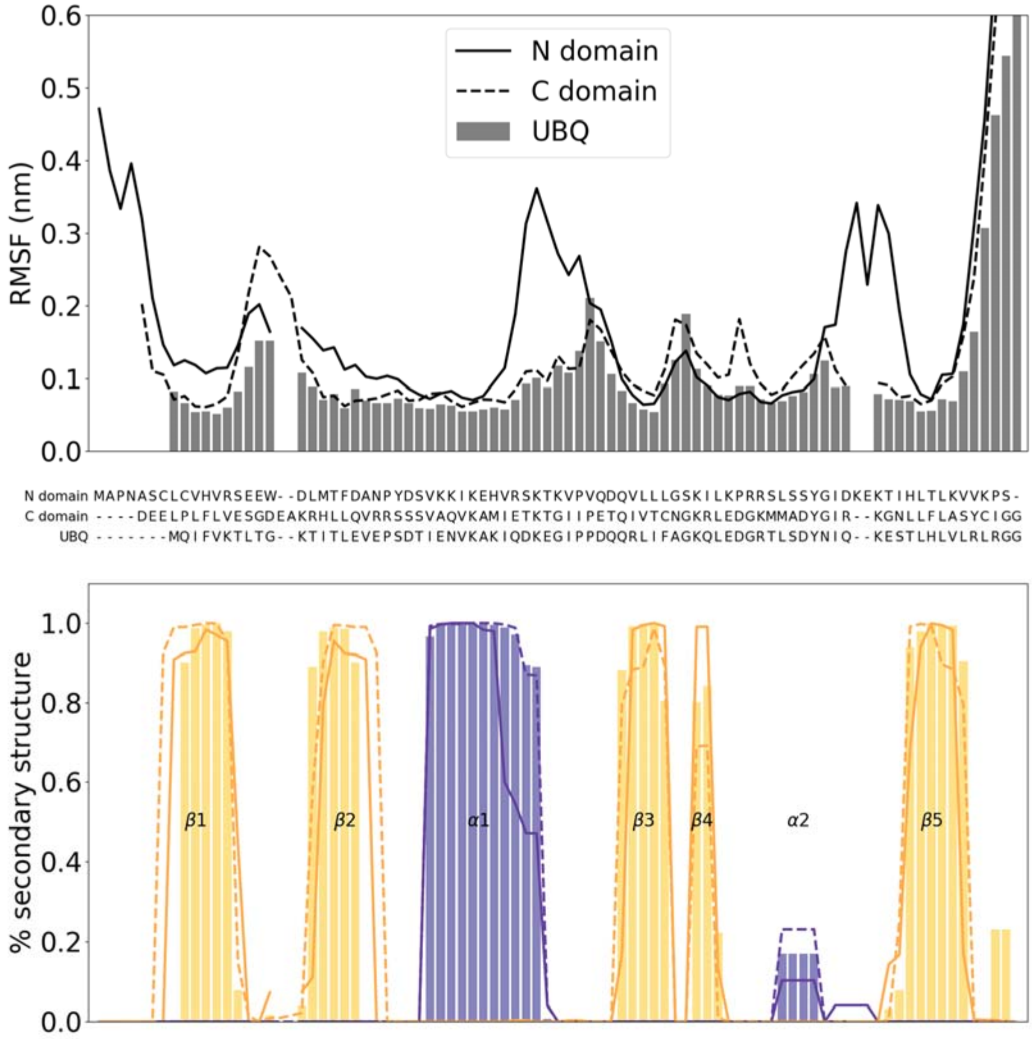

Supplementary Figure 7 The UBDs of FAT10 are less stably folded than Ub. (a) Thermal unfolding data presented as the normalized first derivatives of the temperature dependent

changes in intrinsic tryptophan fluorescence measured at a wavelength of 350 nm ($I_{350\ nm}$) for WT ΔN FAT10 (red), the C0 ΔN FAT10 mutant (green) and ubiquitin (cyan). The first derivative curves ($dI_{350\ nm}$ / dT) are plotted against temperature (T). The respective maxima of the first derivatives correspond to the melting temperatures and are indicated as dashed lines. a.u. denotes arbitrary units. **(b)** Stability of FAT10 backbone. Top panel: Root mean square fluctuation (RMSF) values for the N- and C-domains of FAT10 (WT) in comparison with Ub (PDB-ID: 1UBQ) obtained from MD simulations. Flexibility is increased for loop regions where FAT10 has additional residues compared to Ub. Bottom panel: Percentage of secondary structure appearance during the simulations. The β1, β2 and α1 secondary structure elements are significantly more flexible in the N-domain. All simulations were performed with positive histidine side chains.

a

Figure 3b

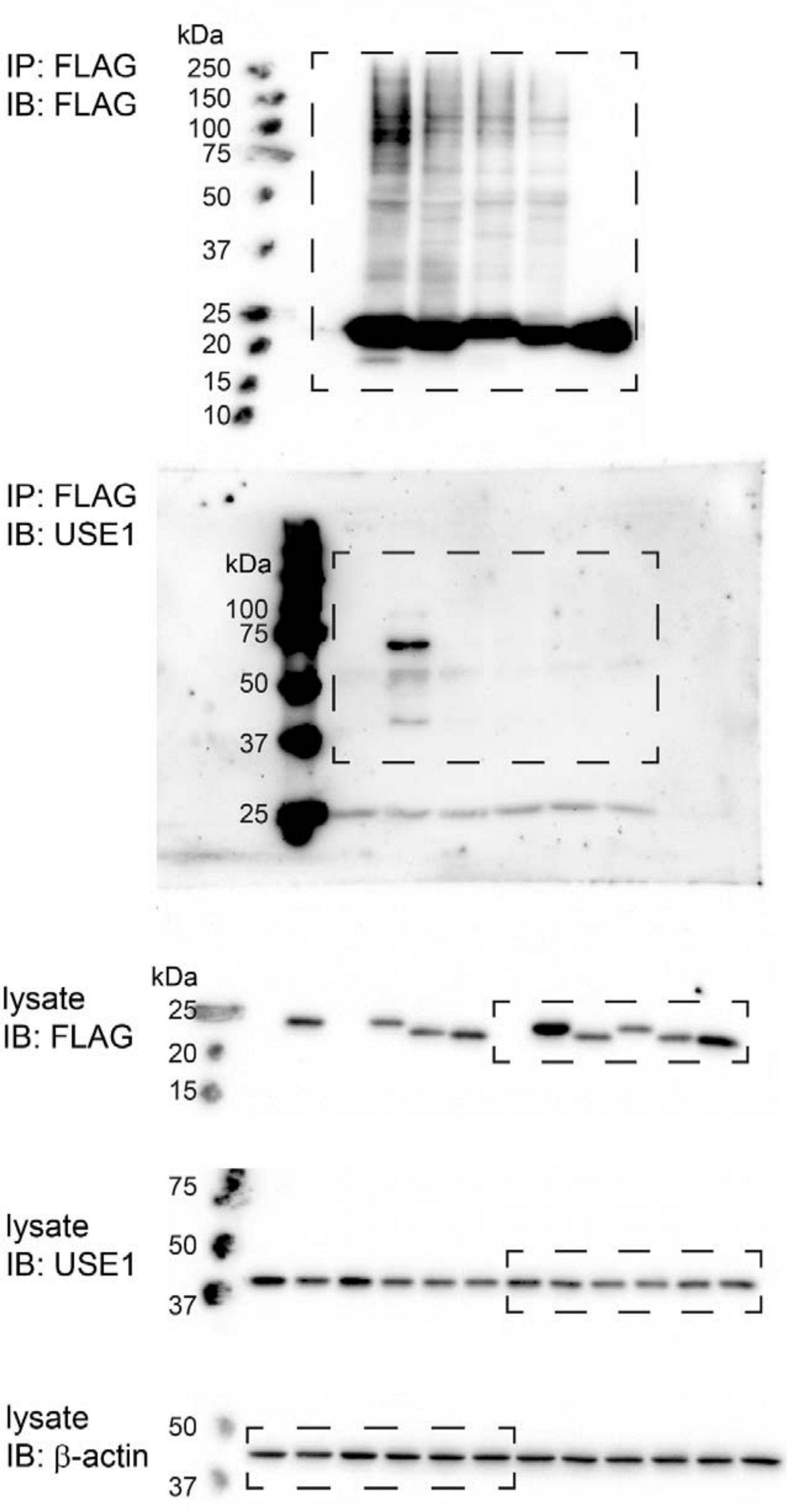
IP: FLAG
IB: FLAG
kDa
250
150
100
75
50
37
25
20
15
10
IP: FLAG
IB: USE1
kDa
100
75
50
37
25
lysate
IB: FLAG
kDa
25
20
15
lysate
IB: USE1
75
50
37
lysate
IB: β-actin
50
37

b

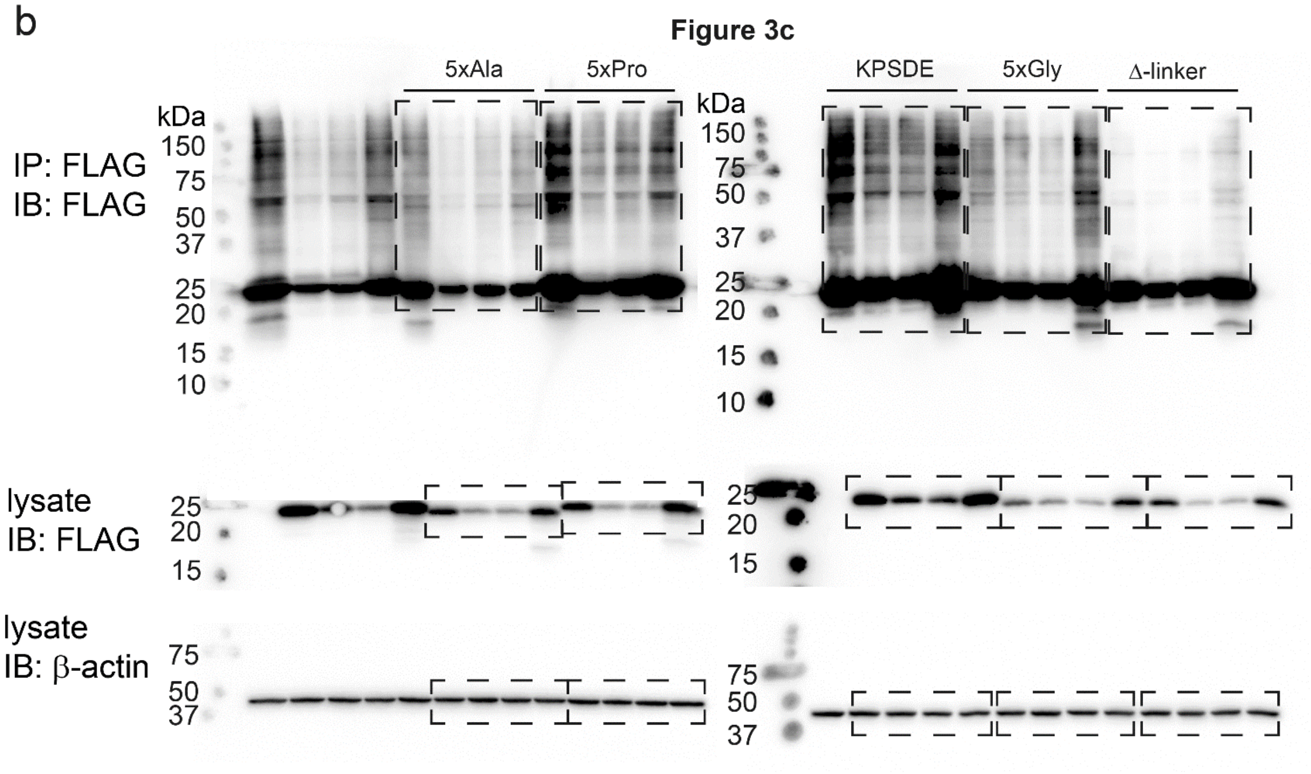
Figure 3c
5xAla
5xPro
KPSDE
5xGly
Δ-linker
kDa
150
75
50
37
25
20
15
10
IP: FLAG
IB: FLAG
lysate
IB: FLAG
lysate
IB: β-actin

C

Figure 4c

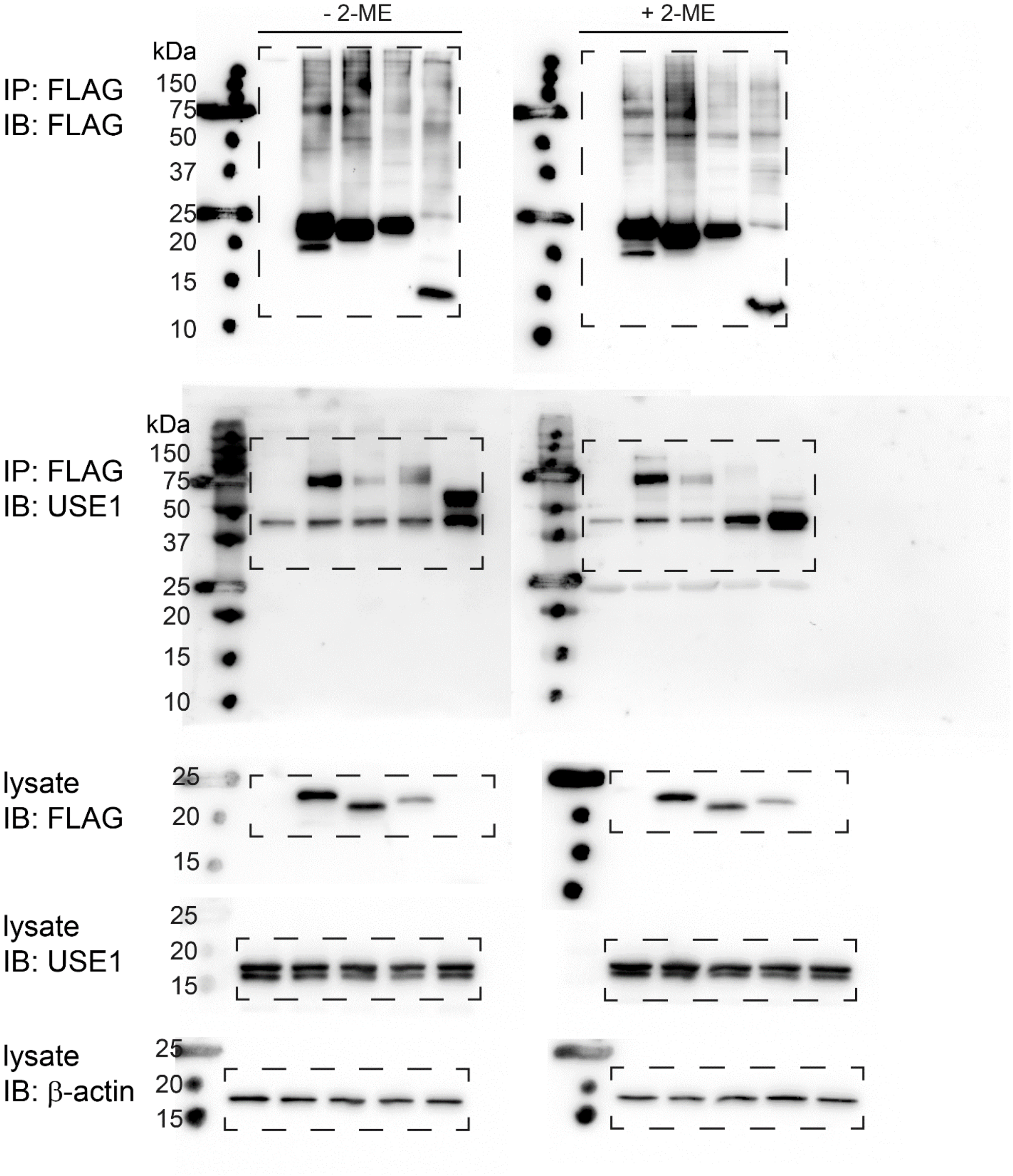

- 2-ME
+ 2-ME
kDa
IP: FLAG
IB: FLAG
150
75
50
37
25
20
15
10
kDa
IP: FLAG
IB: USE1
150
75
50
37
25
20
15
10
lysate
IB: FLAG
25
20
15
lysate
IB: USE1
25
20
15
lysate
IB: β-actin
25
20
15

d

Figure 4d

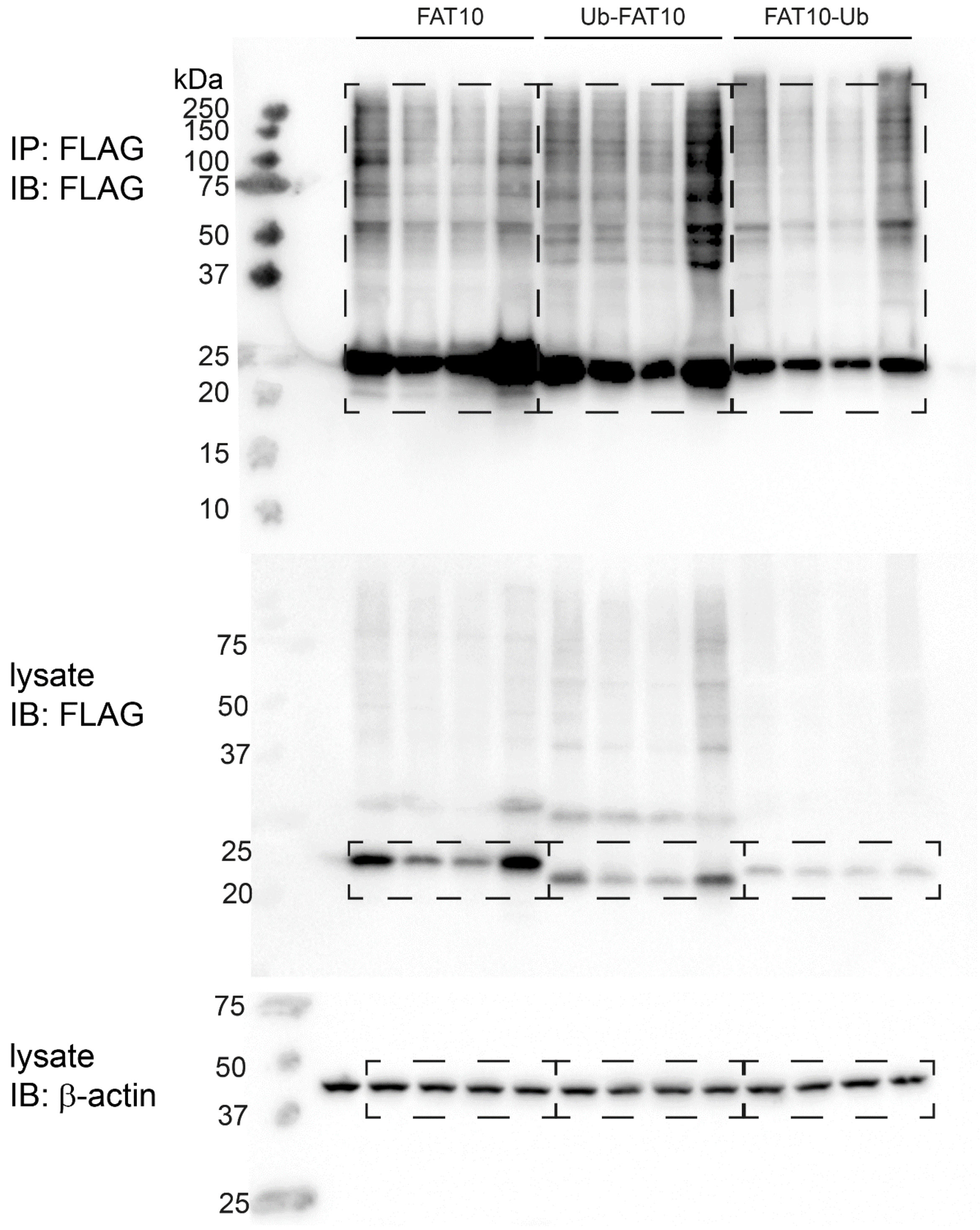

FAT10
Ub-FAT10
FAT10-Ub
kDa
250
150
100
75
50
37
25
20
15
10
IP: FLAG
IB: FLAG
75
50
37
25
20
lysate
IB: FLAG
75
50
37
25
lysate
IB: β-actin

e

Figure 5a

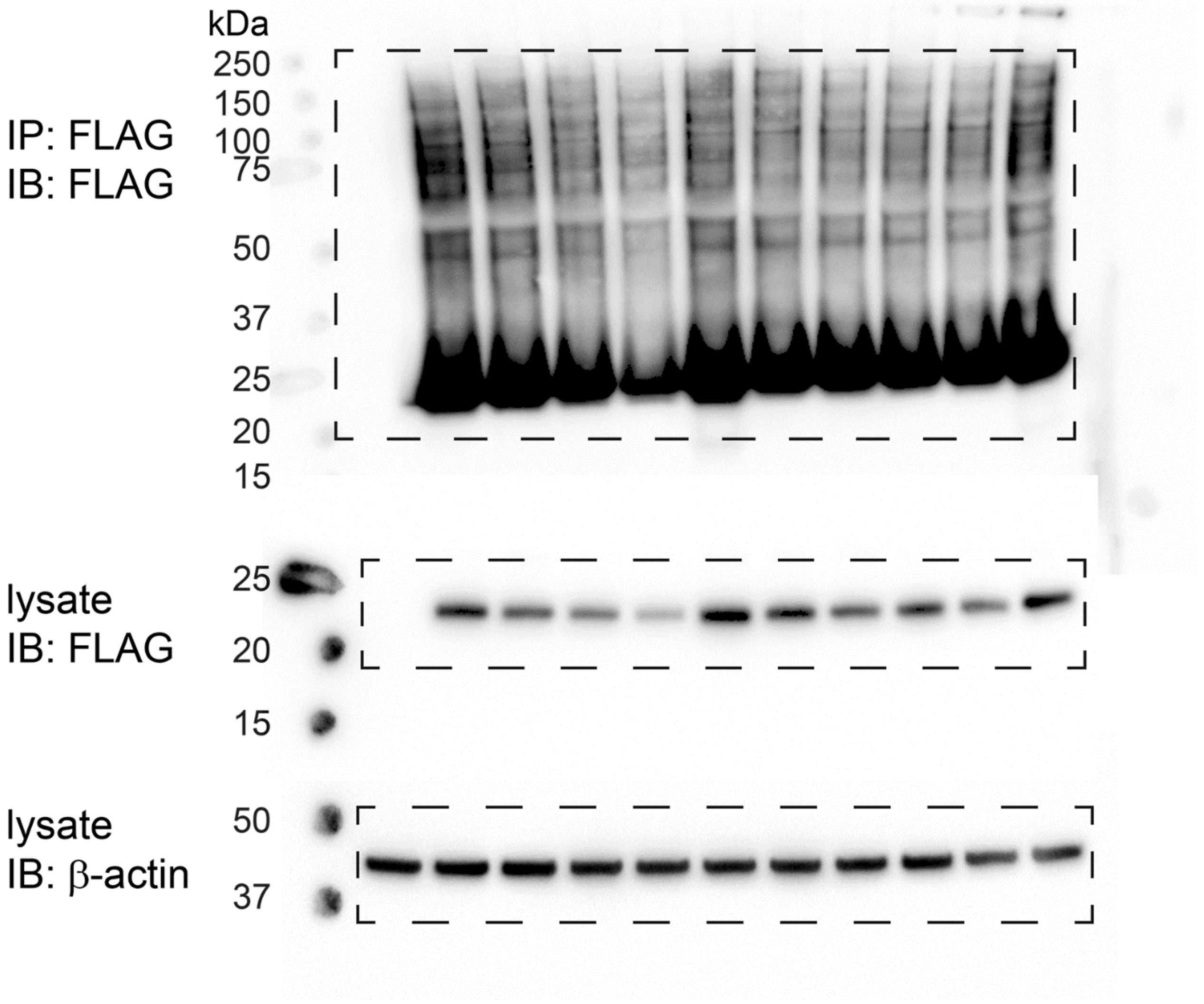
kDa
250
150
100
75
50
37
25
20
15
IP: FLAG
IB: FLAG
25
20
15
lysate
IB: FLAG
50
37
lysate
IB: β-actin

f

Figure 6a

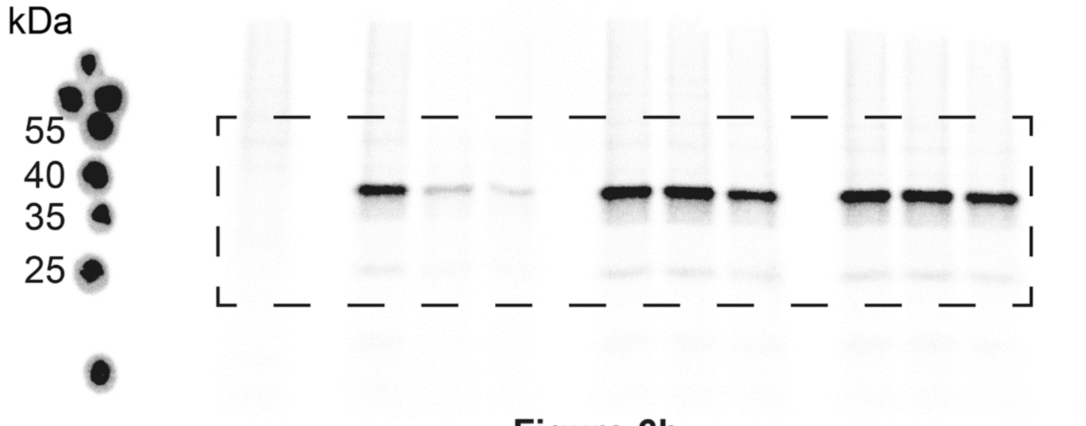

Figure 6b

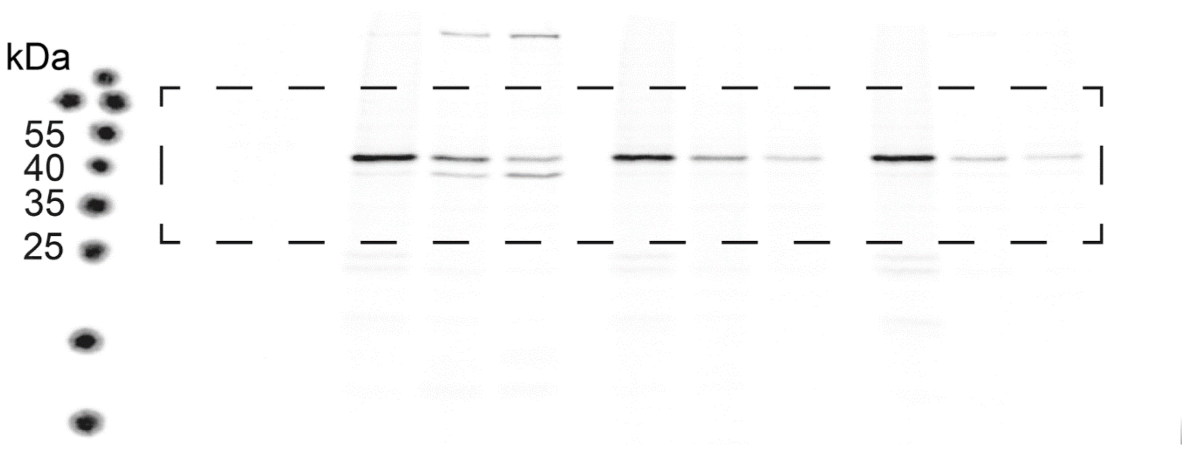

g

Figure 6c

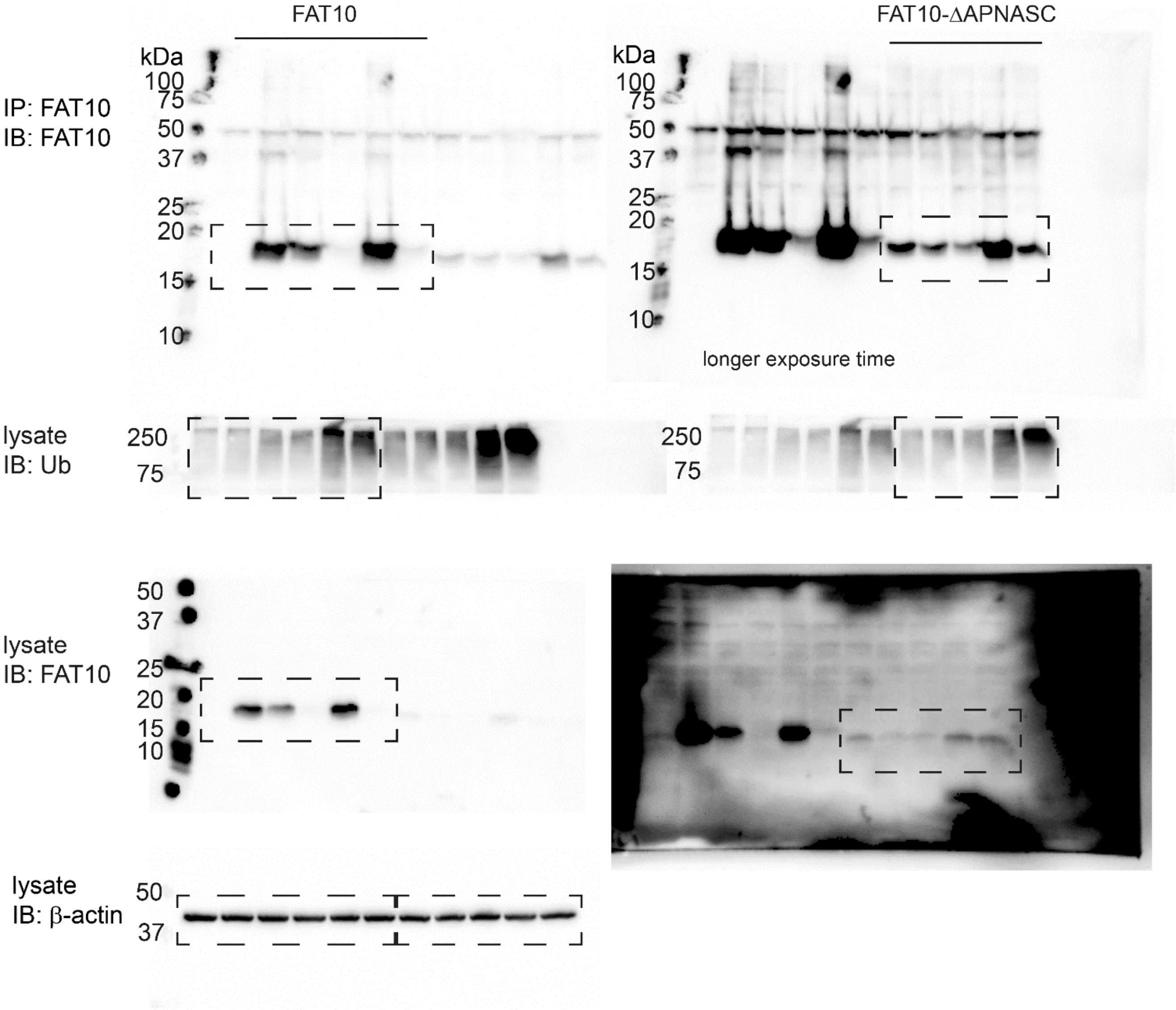
FAT10
FAT10-ΔAPNASC
IP: FAT10
IB: FAT10
kDa
100
75
50
37
25
20
15
10
longer exposure time
lysate
IB: Ub
250
75
lysate
IB: FAT10
lysate
IB: β-actin

h

Supplementary Figure 5b

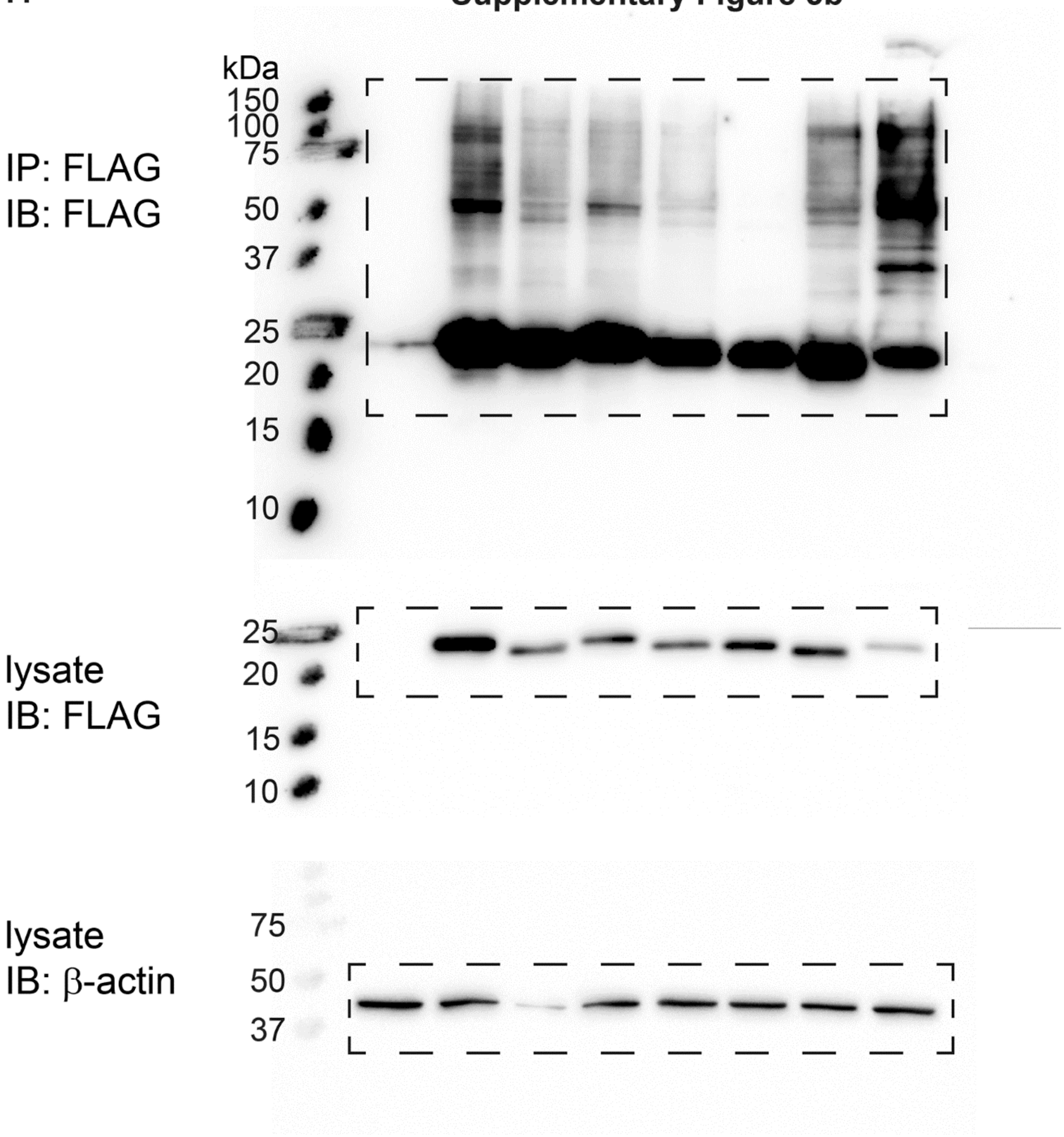

i

Supplementary Figure 6a

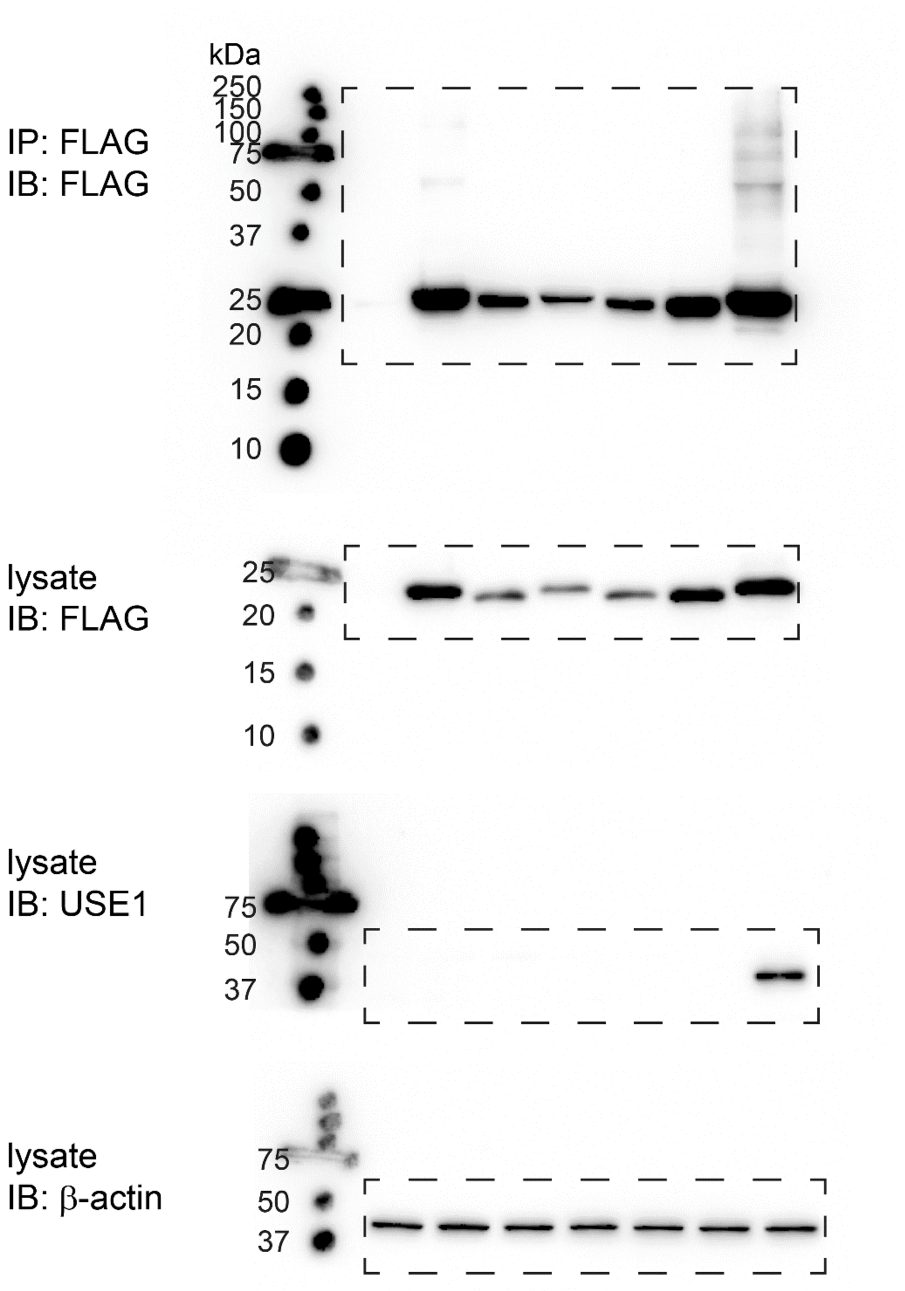

Supplementary Figure 8: Uncropped images of all Western Blots.

(a) Original JPEG data from "Image Lab 4.1." software used to assemble Figure 3b, (b) original JPEG data from "Image Lab 4.1." software used to assemble Figure 3c, (c) original JPEG data from "Image Lab 4.1." software used to assemble Figure 4c, (d) original JPEG data from "Image Lab 4.1." software used to assemble Figure 4d, (e) original JPEG data from "Image Lab 4.1." software used to assemble Figure 5a, (f) original JPEG data from "Image Lab 4.1." software used to assemble Figure 6a and b, (g) original JPEG data from "Image Lab 4.1." software used to assemble Figure 6c, (h) original JPEG data from "Image Lab 4.1." software used to assemble Supplementary Figure 5b, (i) Original JPEG data from "Image Lab 4.1." software used to assemble Supplementary Figure 6a.

Supplementary Table 1 Data collection and refinement statistics (molecular replacement) of the FAT10 N-domain *

**Data collection**	
Space group	R3:H
Cell dimensions	
a, b, c (Å)	116.17, 116.17, 84.72
α, β, γ (°)	90.00, 90.00, 120.00
Resolution (Å)	43.25-1.93 (1.99-1.93) **
R_{merge}	0.0634 (0.74)
$I / \sigma I$	21.4 (2.1)
Completeness (%)	99.31 (93.42)
Redundancy	10.0 (7.0)
**Refinement**	
Resolution (Å)	43.25-1.93
No. reflections	32115 (3051)
R_{work} / R_{free}	0.210/0.233
No. atoms	
Protein	1785
Ligand/ion	30
Water	127
B-factors	
Protein	37.8
Ligand/ion	47.8
Water	41.6
R.m.s. deviations	
Bond lengths (Å)	0.007
Bond angles (°)	1.17

* Number of crystals equals one. **Values in parentheses are for highest-resolution shell.

Supplementary Table 2 NMR and refinement statistics for the 10 Lowest Energy Structures of the FAT10 C-domain

	<SA>*
**NMR distance and dihedral constraints**	
Distance constraints	
Total NOE	3467
Intra-residue	1632
Inter-residue	1835
Sequential ($\|i-j\| = 1$)	777
Medium-range ($2 \le \|i-j\| \le 5$)	385
Long-range ($\|i-j\| \ge 5$)	673
Hydrogen bonds	82
Total dihedral angle restraints	136
ϕ	70
ψ	66
**Structure statistics**	
Violations (mean and s.d.)	
Distance constraints (Å)	0.023 ± 0.009
Dihedral angle constraints (º)	0.531 ± 0.069
Max. dihedral angle violation (º)	3.724
Max. distance constraint violation (Å)	0.485
Deviations from idealized geometry	
Bond lengths (Å)	0.0031 ± 0.0002
Bond angles (º)	0.423 ± 0.012
Impropers (º)	0.370 ± 0.017
Average pairwise r.m.s. deviation** (Å)	
Heavy	0.95 ± 0.11
Backbone	0.27 ± 0.03

*<SA> refers to the ensemble of the 10 structures with the lowest energy out of 250 calculated. Distance constraints were derived from NOE peak intensities using CARA/XEasy[2] and then introduced as unambiguous distances in ARIA[3]. Only unambiguous distance constraints were used.

**Pairwise r.m.s. deviation was calculated among 10 refined structures for residues 87-160.

Supplementary Table S3. Primers and DNA templates used in this study.

Construct number	Construct	Residues (construct)	Vector	Cloning method*	Template	Residues (template)	Mutation	Primer sequences (5' to 3')
1	Cys-free ΔN FAT10	5-165	pETM41	QC	FAT10 C7S, C9S, C134L, C162S in pETM41 (N. Catone)	2-165	APNASS LSVH to GASTLT VH	Forward:<br>TCAGGGCGCCAGTACGCTCACTGTGCAT GTCCGTTCCGAGGAATG<br>Reverse:<br>CATTCCTCGGAACGGACATGCACAGTGA GCGTACTGGCGCCCTGA
2	Cys-free FAT10	1-165	pETM41	QC	Construct #1	5-165	Add MAPN to #1	Forward:<br>CAT GAG CGA GAA TCT TTA TTT TCA GGG CGC CAT GGC GCC GAA CGC GAG TAC GCT CAC TGT GCA TGT CCG TTC C<br>Reverse:<br>GGA ACG GAC ATG CAC AGT GAG CGT ACT CGC GTT CGG CGC CAT GGC GCC CTG AAA ATA AAG ATT CTC GCT CAT G
3	FAT10	1-165	pETM41	QC	Construct #2	1-165	T7C, T9C, L134C, S162C	T7C/T9C forward:<br>CAT GAG CGA GAA TCT TTA TTT TCA GGG CGC CAT GGC GCC GAA CGC GAG TAC GCT CAC TGT GCA TGT CCG TTC C<br>T7C/T9C reverse:<br>GGA ACG GAC ATG CAC AGT GAG CGT ACT CGC GTT CGG CGC CAT GGC GCC CTG AAA ATA AAG ATT CTC GCT CAT G<br>L134C forward:<br>GAA ACC CAG ATT GTG ACT TGC AAT GGA AAG AGA CTG GAA G<br>L134C reverse:<br>CTT CCA GTC TCT TTC CAT TGC AAG TCA CAA TCT GGG TTT C<br>S162C forward:<br>CTTCCTGGCATCTTATTGCATTGGAGGGT GAGGTAC<br>S162C reverse:<br>GTACCTCACCCTCCAATGCAATAAGATG

								CCAGGAAG
4	Cys-free N-domain	8-82	pETM41	RE	Construct 20	8-82	-	Forward:<br>CATGCCATGGGGCTCTCTGTGCATGTCCGTTCC<br>Reverse:<br>CGGGGTACCTCACTTCACCACTTTCAGGGTAAGGTGG
5	Cys-free N-domain	8-82	pETM30	RF	Construct #4	8-82	-	Forward:<br>cga gaatctttcttttcagggcgccatggggctctctgtgcatgtc<br>Reverse:<br>ctg aaagtggtgaagtgaggtaccggatccgaattcgagctccgtc
6	Cys-free N-domain	5-86	pETM30	QC	Construct #5	8-82	LSVH to ASTLTVH	Forward:<br>cag ggc gcc agt acg ctc act gtg cat gtc cgt tcc<br>Reverse:<br>gga acg gac atg cac agt gag cgt act ggc gcc ctg
7	Cys-free C-domain	85-160	pETM10_60	RE	Construct #20	91-160	C134L / add DEELPL to N-terminal part of C-domain	Forward:<br>CATGCCATGGGGTTTCTTGTGGAGTCAGGTGATGAGG<br>Reverse:<br>CGGGGTACCTCAAGATGCCAGGAAGAGTAAGTTGC
8	Cys-free C-domain	85-165	pETM10_60	QC	Construct #7	85-160	add YSIGG to C-term	Forward:<br>GGCAACTTACTCTTCCTGGCATCTTATTCTATTGGAGGGTGAGGTACCGGATCCGAATTCGA<br>Reverse:<br>TCGAATTCGGATCCGGTACCTCACCCTCCAATAGAATAAGATGCCAGGAAGAGTAAGTTGCC
9	RPN10 UIM2	270-301	pRTDuet	RF	pRTDuet	-	-	Forward:<br>ACGACGCTACCAAAACCTACACCGTAACAGAAGGCTCGGGCtttggccgcaccggcctgccggatctgagcagcatgaccgaagaagaacagattgcg<br>Reverse:<br>CGGTATGATCATGATCCGGATCTGCTTAg

								ccaaattccgcgccctgcaggctcatctgcatcgcatacgcaatct gttcttcttcggtcatgctgctc
10	FAT10 5xAla	1-165	pcDNA3.1-His-3xFLAG	SDM	pcDNA3.1-His-3xFLAG-FAT10	1-165	K82A, P83A, S84A, D85A, E86A	Forward: (AA-363) CCATCCACCTTACCCTGAAAGTGGTGGC GGCCGCTGCTGCGGAGCTGCCCTTGTTTC TTGTGG-3` Reverse: (AA-364) CCACAAGAAACAAGGGCAGCTCCGCAGC AGCGGCCGCCACCACTTTCAGGGTAAGG TGGATGG
11	FAT10 5x Pro	1-165	pcDNA3.1-His-3xFLAG	SDM	pcDNA3.1-His-3xFLAG-FAT10	1-165	K82P, P83P, S84P, D85P, E86P	Forward: (AA-361) CCATCCACCTTACCCTGAAAGTGGTGCC GCCCCCTCCTCCGGAGCTGCCCTTGTTTC TTGTGG Reverse (AA-362) CCACAAGAAACAAGGGCAGCTCCGGAG GAGGGGGCGGCACCACTTTCAGGGTAAG GTGGATGG
12	FAT10 5xGly	1-165	pcDNA3.1-His-3xFLAG	SDM	pcDNA3.1-His-3xFLAG-FAT10	1-165	K82G, P83G, S84G, D85G, E86G	Forward: (AA-367) CCATCCACCTTACCCTGAAAGTGGTGGG GGGCGGTGGTGGGGAGCTGCCCTTGTTT CTTGTGG Reverse (AA-368) CCACAAGAAACAAGGGCAGCTCCCCACC ACCGCCCCCCACCACTTTCAGGGTAAGG TGGATGG
13	FAT10 Δ-linker	1-81 and 87-165	pcDNA3.1-His-3xFLAG	SDM	pcDNA3.1-His-3xFLAG-FAT10	1-165	without K82, P83, S84, D85, E86	Forward: (AA-369) GAAGACCATCCACCTTACCCTGAAAGTG GTGGAGCTGCCCTTGTTTCTTGTGGAGTC AGG Reverse (AA-370) CCTGACTCCACAAGAAACAAGGGCAGCT CCACCACTTTCAGGGTAAGGTGGATGGT CTTC
14	FAT10	1-165	pcDNA6.1-myc-His	RE	pcDNA3.1-His-3xFLAG-FAT10	1-165	-	-

15	FAT10 ΔAPNASC	8-165	pcDNA6.1-myc-His	RE	pcDNA3.1-His-3xFLAG-FAT10	8-165	without A2, P3, N4, A5, S6, C7	Forward: (5’-EcoRI-FAT10 dAPNASC fwd) CCGGAATTCATGCTCTGTGTGCATGTC Reverse: (3’-NotI-FAT10 dAPNASC rev) ATAGTTTAGCGGCCGCTCACCCTCCAATACAATAAGATGC
16	FAT10-Ub(K0) (FAT10-Ub)	1-86	pcDNA3.1-His-3xFLAG	SDM	pcDNA3.1-His-3xFLAG-FAT10 (template for SDM); pRK5-HA-Ubiquitin(K0) (template for generation of SDM primers)	1-165	exchange of FAT10-C domain with Ub(K0)	Forward: (AA-371) GACATCGATTACAAGGATGACGATGACAAGGAATTCATGCAGATCTTCGTCAGAACGTTAACCG Reverse: (AA-372) CGACCTTACATCTTGTCTTAAGACTAAGAAAGCCCAGTGATGAGGAGCTGCCCTTGTTTCTTGTGG
17	Ub(K0) ΔGG-FAT10 (Ub-FAT10)	82-165	pcDNA3.1-His-3xFLAG	SDM	pcDNA3.1-His-3xFLAG-FAT10 (template for SDM); pRK5-HA-Ubiquitin(K0) (template for generation of SDM primers)	1-165	exchange of FAT10-N domain with Ub(K0)	Forward: (AA-373) CCACCTTACCCTGAAAGTGGTGAAGCCCAGTGATGAGATGCAGATCTTCGTCAGAACGTTAACCG Reverse: (AA-374) CATCTTGTCTTAAGACTAAGAGGTGGTTGACGCGGCCGCGCTCGCCCAGCACTCGGTGCCG
18	HA-FAT10-(C0)-C134L	1-165	pSUMO	SDM	Construct # 21	1-165	C134L	Forward: CCCTGAAACCCAGATTGTGACTCTGAATGGAAAGAGACTGGAAGATG Reverse: CATCTTCCAGTCTCTTTCCATTCAGAGTCACAATCTGGGTTTCAGGG
19	FAT10-(C0)-C134L	1-165	pcDNA3.1-His-3xFLAG	RE	Construct # 18	1-165	-	Forward: (AA-345) CGCGGAATTCATGGCTCCCAATGCTTCCAGCCTCTC-3`

								Reverse: (AA-346) GGCATCTTATTCTATTGGAGGGTGACGC GGCCGCGCTCGCC
20	FAT10-(C0)	1-165	pSUMO	SDM	pSUMO-FAT10 Aichem et al 2014	1-165	C7S, C9S, C134S, C162S	Forward C7S, C9S: CTCCCAATGCTTCCAGCCTCTCTGTGCAT GTCCGTT Forward C134S: TCCCTGAAACCCAGATTGTGACTAGCAA TGGAAAGA Forward C162S: ACTTACTCTTCCTGGCATCTTATTCTATT GGAGGGTGAC
21	HA-FAT10-(C0)	1-165	pSUMO	RE	Construct # 22	16-181	-	Forward: CCTGGCATCTTATTCTATTGGAGGTTAGC TCGAGCACCAC Reverse: GTGGTGCTCGAGCTAACCTCCAATAGAA TAAGATGCCAGG
22	HA-FAT10-(C0)	1-164	pTYB2	SDM	pTYB2-HA-FAT10<br><br>Hemelaar et al. 2004	16-181	C7S, C9S, C134S, C162S	Forward C7S, C9S: CTCCCAATGCTTCCAGCCTCTCTGTGCAT GTCCGTT Forward C134S: TCCCTGAAACCCAGATTGTGACTAGCAA TGGAAAGA Forward C162S: ACTTACTCTTCCTGGCATCTTATTCTATT GGAGGGTGAC
23	Cys-free C-domain C134L	85-160	pETM10_60	SDM	Construct #7	85-160	C134L	Forward: ccctgaaacccagattgtgactctgaatggaaagagactggaagat g Reverse: catcttccagtctctttccattcagagtcacaatctgggtttcaggg

*QC: QuikChange; RF: Restriction Free; SDM: site-directed mutagenesis; RE: restriction enzymes

Supplementary References

1. Theng, S.S. *et al.* Disruption of FAT10-MAD2 binding inhibits tumor progression. *Proc. Natl. Acad. Sci. U S A* **111**, E5282-E5291 (2014).
2. Bartels, C., Xia, T.H., Billeter, M., Guntert, P. & Wuthrich, K. The program XEASY for computer-supported NMR spectral analysis of biological macromolecules. *J. Biomol. NMR* **6**, 1-10 (1995).
3. Nilges, M. & O'Donoghue, S.I. Ambiguous NOEs and automated NOE assignment. *Prog. Nucl. Magnet. Res. Spectr.* **32**, 107-139 (1998).
4. Laskowski, R.A., Rullmannn, J.A., MacArthur, M.W., Kaptein, R. & Thornton, J.M. AQUA and PROCHECK-NMR: programs for checking the quality of protein structures solved by NMR. *J. Biomol. NMR* **8**, 477-486 (1996).
